# Parametric bionic hand-inspired optimization of femoral condylar prosthesis attachment surfaces

**DOI:** 10.3389/fbioe.2025.1656421

**Published:** 2025-10-29

**Authors:** Lin Wang, Wen Zhou, Hui Sun, Shuqi Lian

**Affiliations:** School of Medical Information and Engineering, Xuzhou Medical University, Xuzhou, China

**Keywords:** femoral condylar prosthesis, attachment surface, bionic hand-inspired structure, parametric design, knee arthroplasty

## Abstract

Traditional femoral condylar prosthesis attachment surfaces often lack adequate anatomical conformity, resulting in clinical complications such as prosthesis loosening and stress shielding. Inspired by the multi-level curvature adaptation observed in the palmar-phalangeal hierarchy, this study introduces a novel bionic hand-inspired design methodology to enhance the adaptability of prosthesis attachment surfaces. Unlike conventional biomimetic approaches that primarily focus on replicating macroscopic shapes, our method transforms the functional hierarchy of phalange-palm interactions into a parametric design system, enabling dynamic curvature control to improve the fit of the prosthesis to the condylar resection surface. The proposed framework encompasses: (1) constructing bionic finger contour feature lines based on critical anatomical landmarks, (2) parameterizing the bionic fitting surface through bending and dimensional parameters, and (3) projecting this surface onto the femoral condyle to generate the attachment surface. Experimental validation across parametric variations (n = 4 groups) confirmed that the optimized bionic structure offers superior editability, anatomical adaptability, and a significantly improved fit, as evidenced by a Hausdorff distance of 0.29 mm. This approach simplifies the design process compared to conventional CAD-based methods while providing clinically adaptable parameters. The methodology demonstrates potential for application to a broader range of orthopedic implant designs where anatomical conformity is critical.

## 1 Introduction

The femoral condylar prosthesis, a pivotal component in knee prostheses, plays an indispensable role in total knee replacement (TKR) procedures (Thomas, 2021; Carr et al., 2012; Asseln et al., 2021; Kubicek et al., 2019; Koh et al., 2020; Frysz et al., 2020). The design of the prosthesis significantly influences postoperative functionality and patient recovery (Shi et al., 2015; Twiggs et al., 2018). Specifically, the attachment surface, which substitutes for the native femoral condyle, demands precise anatomical alignment to ensure optimal rehabilitation outcomes and minimize complications such as prosthesis loosening and compromised vascular supply.

Currently, prosthesis design predominantly relies on computer-aided design (CAD) technology. Commercial software packages (e.g., CATIA, SolidWorks) enable customized prosthesis construction through labor-intensive point/line/surface manipulations. Three fundamental limitations impede clinical translation: (1) Static geometric mismatch: Manual/CAD-based approaches (e.g., SolidWorks, CATIA) yield inadequate anatomical conformity (associated with a 20.3% long-term loosening rate (Sharkey et al., 2014)), stemming from oversimplified curvature modeling that ignores dynamic joint kinematics during flexion/extension (Abdel et al., 2015). (2) Dynamic functional disconnect: Additive manufacturing perpetuates design-phase inaccuracies, causing interface stress concentration during motion (Liu and Shin, 2019). Similarly, computational methods (e.g., Liu’s data-driven pipeline (Liu et al., 2022)) prioritize biomechanical simulation over real-time intraoperative adaptability. (3) Clinical efficiency barrier: Parametric frameworks lack anatomically meaningful semantic controls (requiring >6 h per case (Harrysson et al., 2014)), while emerging robotic solutions (e.g., Herr’s emulators (Herr et al., 2023)) emphasize dynamic mobility at the cost of static morphological precision.

The growing clinical demand for patient-specific prostheses has been extensively documented (Lee et al., 2020; P et al., 2022), particularly among Asian populations, who exhibit distinct femoral morphologies (Fan et al., 2017). The precision of fitting is paramount in determining the clinical success of TKR. Enhanced fitting accuracy can substantially reduce postoperative complications and elevate patient satisfaction (Brinkmann and Fitz, 2021), and may also contribute to more effective postoperative rehabilitation, as optimized conservative treatment strategies are crucial for functional recovery (Mascia et al., 2025). However, achieving adaptable attachment surfaces remains a challenge due to limitations in both universality and precision of fit. Two key challenges persist: (1) the rapid generation of surfaces with variable shapes through parameter adjustments, and (2) effective local shape customization via detailed feature-level control. These difficulties stem from the unique and highly variable morphology of the femoral condyle (Bonnin et al., 2016).

**FIGURE 1:**
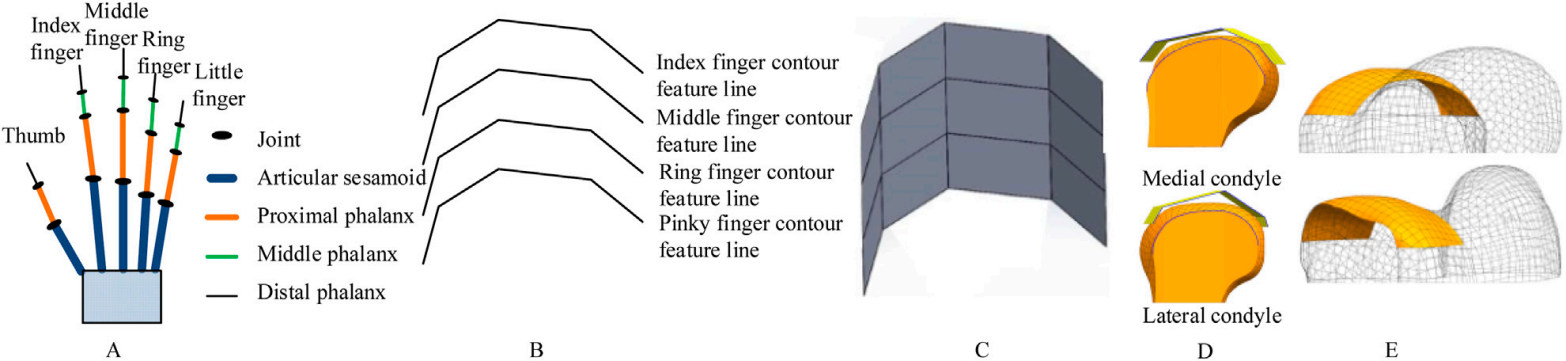
Schematic of bionic hand-inspired structure generation. **(A)** Human hand structure, encompassing the thumb, index finger, middle finger, ring finger, and little finger. Each finger consists of the articular sesamoid, proximal phalanx, middle phalanx, and distal phalanx. **(B)** Bionic finger contour feature lines, including the index finger, middle finger, ring finger, and little finger contour feature lines. **(C)** Bionic fitting surface, constructed based on the finger contour feature lines in Figure 1B and subsequent filling. **(D)** Projection of the bionic fitting surface onto the medial condyle of the femur. **(E)** Attachment surface.

Our proposed solution is inspired by the remarkable adaptability of the human hand (Figure 1A). The multi-joint phalangeal structure of the human hand enables it to conform effectively to objects of various shapes (Wang et al., 2023). This bionic design aligns with an emerging trend—shifting from mere shape replication to the integration of functional biomechanical principles (Baek et al., 2023). Specifically, the layered curvature control mechanism of the human hand offers a promising approach for adapting to complex anatomical surfaces (Bai et al., 2024).

**FIGURE 2:**
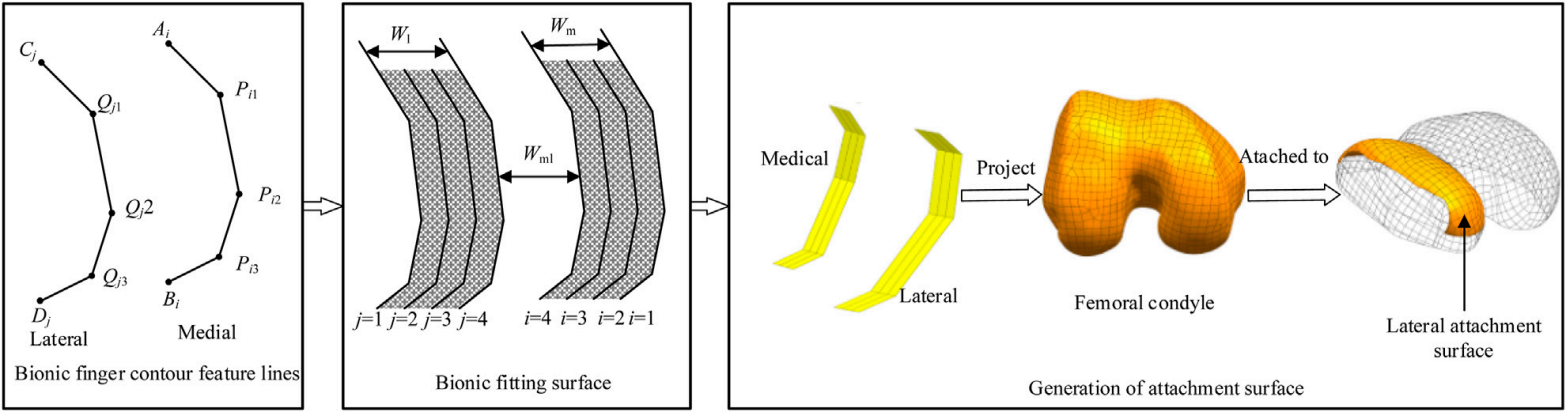
Research framework with bionic-functional mapping. ① Level 1 joint (*Q*_*j*1_, *P*_*i*1_) corresponds to the femoral condyle apex, controlling longitudinal curvature. ② Level 2 joint (*Q*_*j*2_, *P*_*i*2_) matches the intercondylar fossa edge, facilitating transverse bending adjustment. ③ Level 3 joint (*Q*_*j*3_, *P*_*i*3_) optimizes the condylar base curvature, enabling dynamic conformity. The research framework depicts the construction of bionic finger contour feature lines, which consist of lateral and medial lines, each comprising four feature lines. The bionic fitting surface is constructed based on these feature lines and subsequent filling processes. The femoral condylar prosthetic attachment surface is then generated through projection, segmentation, and reconstruction techniques, aligning the medial lines with the medial surface and the lateral lines with the lateral surface.

**FIGURE 3:**
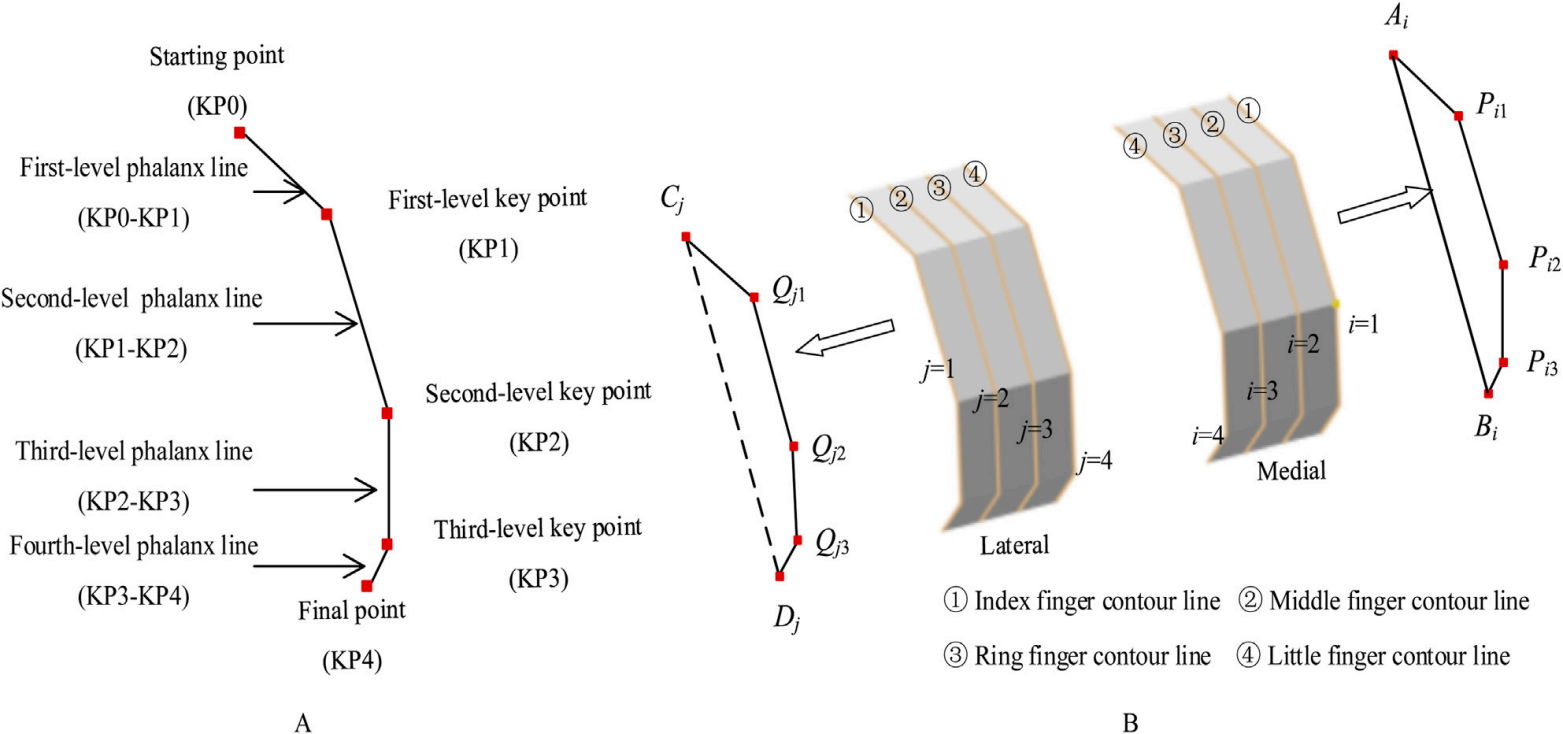
Bionic finger contour feature lines. **(A)** Composition of a bionic finger contour feature line, constructed from key points including the starting point, first-level key point, second-level key point, third-level key point, and final point. These key points generate first-level, second-level, third-level, and fourth-level phalanx lines. **(B)** Medial and lateral bionic finger contour feature lines, consisting of index, middle, ring, and little finger contour feature lines. The medial lines are sequentially numbered 1 to 4 from inside to outside (*A*_*i*_, *P*_*i*1_, *P*_*i*2_, *P*_*i*3_, *B*_*i*_), while the lateral lines are numbered 1 to 4 from outside to inside (*C*_*j*_, *Q*_*j*1_, *Q*_*j*2_, *Q*_*j*3_, *D*_*j*_).

Building upon our research group’s prior work in bone morphological feature extraction (Wang et al., 2021; Wang et al., 2016)and implant construction (Wang et al., 2022), as well as the development of a semantic feature parameter system for bone plate design (Wang et al., 2017), we propose a novel method for designing femoral condylar prosthesis attachment surfaces inspired by bionic hand-inspired structure. Our methodology comprises three pivotal steps: (1) Constructing bionic finger contour feature lines using anatomical key points (Figure 1B), (2) Generating adjustable bionic fitting surfaces (Figure 1C), and (3) Creating attachment surfaces through projection (Figures 1D,E). The bionic fitting surface can be efficiently tailored through bending and size parameters, achieving a balance between simplicity and clinical adaptability.

This research contributes to the advancement of knee prosthesis design by: (1) Offering a flexible parametric framework that complements existing methodologies, (2) Establishing scientifically validated surface design principles, and (3) Delivering clinically significant enhancements in anatomical fitting. The subsequent sections delineate our methodology (Section 2), experimental implementation (Section 3), and conclusions along with future research directions (Section 4).

This bionic design aligns with an emerging trend—shifting from mere shape replication to the integration of functional biomechanical principles (Baek et al., 2023). Specifically, the layered curvature control mechanism of the human hand, which enables conformal grasping of irregular objects (Wang et al., 2023; Bai et al., 2024), offers a functional analogy for designing prosthetic surfaces that can dynamically adapt to the complex and variable morphology of the femoral condyle. The proposed method does not seek to replicate the exact shape of a hand, but rather to translate its underlying principle of multi-level, parametric curvature adaptation into a practical design framework.

## 2 Materials and methods

### 2.1 Overview of the proposed framework

The methodology proposed in this study was validated using clinical computed tomography (CT) data with ethical approval obtained from the Ethics Committee of Xuzhou Medical University. All procedures adhered to the principles outlined in the Declaration of Helsinki. CT images of patients were acquired under the supervision of a clinician, acknowledging the inherent individual differences in femoral condylar morphology. To demonstrate the feasibility of the proposed method while acknowledging anatomical diversity, a representative case—a 50-year-old Han Chinese male with a height of 175 cm—was selected for analysis. The CT scanning was performed using a Light Speed VCT helix scanner manufactured by GE, with the following primary parameters: tube voltage set at 120 kV, tube current at 300 mA, layer thickness of 0.6 mm, layer spacing of 5.0 mm, and a scanning duration of 1.5 s.

To improve the design quality of the femoral condylar prosthesis, we propose a novel design method for its attachment surface, inspired by the bionic hand-inspired structure, as shown in Figure 2. The main steps of this framework are as follows:

Step 1: Construction of Bionic Finger Contour Feature Lines

Bionic finger contour feature lines are constructed based on key anatomical landmarks. These lines encompass both lateral and medial components, each meticulously crafted using key points corresponding to the joints of the human finger structure.

Step 2: Development of Bionic Fitting Surface

The bionic fitting surface is generated based on the bionic finger contour feature lines derived in Step 1. This surface, similarly divided into lateral and medial components, is parameterized to facilitate straightforward modification and editing. The parameters governing this surface include bending and size parameters, which offer flexibility in design adjustments.

Step 3: Projection and Reconstruction of Attachment Surface

The bionic adaptation surface undergoes orthogonal projection onto the femoral condylar anatomy. This is followed by advanced segmentation and surface reconstruction algorithms, resulting in a morphologically optimized attachment interface that ensures precise anatomical conformity.

This bionic design principle is translated into the prosthesis through a hierarchical mapping of the phalangeal architecture onto the femoral condylar surface. This mapping establishes three distinct functional correspondences: the Level 1 joint (*Q*_*j*1_, *P*_*i*1_) corresponds to the femoral condyle apex to control longitudinal curvature; the Level 2 joint (*Q*_*j*2_, *P*_*i*2_) matches the intercondylar fossa edge to facilitate transverse bending adjustment; and the Level 3 joint (*Q*_*j*3_, *P*_*i*3_) optimizes the condylar base curvature, enabling dynamic prosthesis conformity.

This parametric mapping facilitates a graded adaptation mechanism. By adjusting the bionic fitting parameters (including bending and size parameters, as detailed in Table 1), the fitting surface can be tailored to a wide range of anatomical morphologies.

**TABLE 1:** Bending and size parameters of the bionic fitting surface (Units: mm, °; *i* = 1, 2, 3, 4; *j* = 1, 2, 3, 4).

Group	Bending parameters			Length parameters				Width parameters
	*α*_*i*1_	*α*_*i*2_	*α*_*i*3_	*M*_*i*1_	*M*_*i*2_	*M*_*i*3_	*M*_*i*4_	*W*_*m*_
	*β*_*j*1_	*β*_*j*2_	*β*_*j*3_	*L*_*i*1_	*L*_*i*2_	*L*_*i*3_	*L*_*i*4_	*W*_*l*_
1	140.01	122.45	143.48	10.74	30.86	24.66	9.15	21.20
	133.86	109.67	157.53	16.99	26.09	18.39	12.11	22.80
2	143.91	141.21	144.32	10.74	31.92	21.17	10.74	21.34
	145.87	153.05	129.17	10.75	29.86	20.22	8.87	23.30
3	138.79	124.03	141.58	10.74	32.86	20.58	10.74	21.61
	134.20	129.96	141.37	8.86	31.78	22.14	6.87	24.62
4	133.20	124.03	138.19	10.74	32.85	20.57	10.74	21.61
	144.10	151.29	129.17	10.74	28.52	20.22	8.86	23.10

### 2.2 Bionic finger contour feature lines

#### 2.2.1 Anatomical basis

Feature lines serve as the foundation for surface modeling, directly influencing the surface shape in orthopedic implant design (He et al., 2014). This approach integrates anatomical features (points, curves, and surfaces) while circumventing the labor-intensive, bottom-up design process. Through parametric design, the implant geometry can be dynamically adjusted to match individual patient anatomy, thereby enhancing compatibility (Li et al., 2023). The resulting surface morphology depends on the configuration of these feature lines and the generation algorithm. The bionic finger contour comprises medial and lateral lines, interconnected by a parametric control mechanism inspired by the functional hierarchy of the metacarpophalangeal structure. These connections ensure curvature adaptability and improve anatomical compatibility, addressing key limitations of traditional femoral condylar prosthesis design processes (Sun et al., 2024). Projecting the bionic finger contour onto the femoral condyle generates an attachment surface with excellent editability and significantly improved fit.

#### 2.2.2 Key point specification

The strategic placement of bionic finger key points is critical, with each point corresponding to specific anatomical landmarks (Figure 3A). These key points encompass a starting point, first-level key point, second-level key point, third-level key point, and final point. The starting point and the first-level key point constitute the first-level phalanx line, corresponding to the articular sesamoid in the hand structure. Subsequent phalanx lines are similarly defined, with the first-level key point and second-level key point forming the second-level phalanx line (proximal phalanx), the second-level key point and third-level key point forming the third-level phalanx line (middle phalanx), and the third-level key point and the final point forming the fourth-level phalanx line (distal phalanx).

To ensure a high degree of fit between the femoral condylar prosthesis attachment surface and the recipient, the bionic hand-inspired structure is simplified into a surface composed of four finger contour lines. As illustrated in Figure 3B, these lines include the index finger contour line, middle finger contour line, ring finger contour line, and little finger contour line. Further details are provided in Sections 2.2.3 and 2.2.4.

#### 2.2.3 Medial contour construction

For the medial bionic hand-inspired structure, the finger contour feature lines, from inside to outside, are the index finger contour feature line (*i* = 1), middle finger contour feature line (*i* = 2), ring finger contour feature line (*i* = 3), and little finger contour feature line (*i* = 4). The key points for constructing each finger contour are the starting point *A*_*i*_, first-level key point *P*_*i*1_, second-level key point *P*_*i*2_, third-level key point *P*_*i*3_, and final point *B*_*i*_. The determination of *P*_*i*1_, *P*_*i*2_, and *P*_*i*3_ is as follows: *P*_*i*1_ is positioned at the same height as *M*_high_, the highest point of the medial femoral condyle, and extends along the negative direction of the coronal axis. The distance between *P*_*i*1_ and *M*_medial_ is denoted as *H*_*i*m1_. *P*_*i2*_ is situated at the same height as *M*_medial_, the most medial point of the medial femoral condyle, and extends along the negative direction of the coronal axis, with the distance between *P*_*i2*_ and *M*_medial_ denoted as *H*_*i*m2_. *P*_*i*3_ is located at the same height as *M*_low_, the most medial point of the medial femoral condyle, and extends along the negative direction of the coronal axis, with the distance between *P*_*i*3_ and *M*_medial_ recorded as *H*_*i*m3_. The distances *H*_*i*m1_, *H*_*i*m2_, and *H*_*i*m3_ are calculated using Equation 1.

$$\left\{\begin{array}{c} H_{im1}=\frac{\left(i‐1\right)\times \left|\vec{M_{medial\;}M_{lateral}}\right|}{3}\times \delta_{1}\\ H_{im2}=\frac{\left(i‐1\right)\times \left|\vec{L_{medial}\;L_{lateral}}\right|}{3}\times \delta_{2}\\ H_{im3}=\frac{\left(i‐1\right)\times \left|\vec{L_{medial}\;L_{lateral}}\right|}{3}\times \delta_{3}\; \end{array}\right.$$ (1)

where *M*_medial_ represents the most medial point of the medial femoral condyle, and *M*_lateral_ represents the most lateral point of the medial femoral condyle. These coefficients ( $\delta_{1}$ - $\delta_{3}$ , $\varepsilon_{1}$ - $\varepsilon_{3}$ ) were empirically calibrated from an analysis of 20 CT scans representing diverse femoral morphologies (as described in Section 2.1), ensuring population-level adaptability to anatomical variations such as Asian-specific condylar dimensions (Fan et al., 2017). The initial values were refined through an iterative process to minimize the average Hausdorff distance across the calibration set.

#### 2.2.4 Lateral contour construction

Similarly, for the lateral bionic hand-inspired structure, the finger contour lines, from outside to inside, are the index finger contour (*j* = 1), middle finger contour (*j* = 2), ring finger contour (*j* = 3), and little finger contour (*j* = 4). The key points for constructing each finger contour of the lateral bionic hand-inspired structure are the starting point *C*_*j*_, first-level key point *Q*_*j*1_, second-level key point *Q*_*j*2_, third-level key point *Q*_*j*3_, and final point *D*_*j*_. *Q*_*j*1_ is positioned at the same height as *L*_high_, the highest point of the lateral femoral condyle, and extends along the positive direction of the coronal axis. The distance between *Q*_*j*1_ and *L*_lateral_ is denoted as *H*_*j*l1_. *Q*_*j*2_ is situated at the same height as *L*_medial_, the highest point of the lateral femoral condyle, and extends along the positive direction of the coronal axis, with the distance between *Q*_*j*2_ and *L*_medial_ denoted as *H*_*j*l2_. *Q*_*j*3_ is located at the same height as *L*_low_, the most lateral point of the lateral femoral condyle, and extends along the positive direction of the coronal axis, with the distance between *Q*_*j*3_ and *L*_medial_ recorded as *H*_*j*l3_. The distances *H*_*j*l1_, *H*_*j*l2_, and *H*_*j*l3_ are calculated using Equation 2.

$$\left\{\begin{array}{c} H_{jl1}=\frac{\left(j‐1\right)\times \left|\vec{L_{medial}\;L_{lateral}}\right|}{3}\times \varepsilon_{1\;}\\ H_{jl2}=\frac{\left(j‐1\right)\times \left|\vec{L_{medial}\;L_{lateral}}\right|}{3}\times \varepsilon_{2\;}\\ H_{jl3}=\frac{\left(j‐1\right)\times \left|\vec{L_{medial}\;L_{lateral}}\right|}{3}\times \varepsilon_{3}\; \end{array}\right.$$ (2)

Here, *L*_medial_ represents the most medial point of the lateral femoral condyle, and *L*_lateral_ represents the most lateral point of the lateral femoral condyle. The coefficients ε _1_, ε _2_ and ε _3_ are empirical values obtained from an analysis of a dataset of femur samples. The selection of *A*_*i*_ and *B*_*i*_ is based on the osteotomy *d*_0_ performed by orthopedic surgeons, satisfying the conditions: $\left\{\begin{array}{c} \left|\vec{P_{i1}A_{i}\;}\right|=d_{0}\\ \left|\vec{P_{i3}B_{i}}\right|=d_{0} \end{array}\right.$ and $\left\{\begin{array}{c} \left|\vec{Q_{j1}\;C_{j}}\right|=d_{0}\\ \left|\vec{Q_{j3}\;D_{j}}\right|=d_{0} \end{array}\right.$ .

#### 2.2.5 Determination of adjustment coefficients (*δ* and *ε*)

The adjustment coefficients (*δ* and *ε*) are dimensionless scaling factors that translate anatomical dimensions of the femoral condyle (e.g., |*M*_medial_ *M*_lateral_|) into offsets (*H*_*i*m1_, *H*_*i*m2_, etc.) for positioning the bionic key points. These coefficients are essential for ensuring the generated contour feature lines are anatomically proportional and adaptable to population variations. Since they define the proportional relationship between bone morphology and the bionic model, the coefficients cannot be derived from first principles and must be empirically calibrated using a representative anatomical dataset. The calibration aimed to optimize anatomical fit, quantified by the Hausdorff distance between the resulting attachment surface and the native condylar surface.

The calibration procedure used a set of femur CT scans with diverse morphological characteristics as the calibration set. The average Hausdorff distance between the prosthesis attachment surface—generated from a candidate set of coefficients—and the native condylar surface was defined as the objective function to minimize. The Nelder-Mead simplex algorithm was employed for iterative optimization, which converged to values of *δ*_1_–*δ*_3_ and *ε*_1_–*ε*_3_ approximately equal to 0.76.

This data-driven calibration ensures the parametric model captures essential statistical relationships between bone size and the curvature distribution required for a conformal fit. The value 0.76 serves as a population-average starting point; for patient-specific applications, these coefficients can be further fine-tuned using individual CT data to achieve superior fit.

### 2.3 Parametric bionic fitting surface design

Semantic feature parameters are medically meaningful parameters that facilitate high-level design operations. They encompass both global parameters, which characterize the overall model geometry (e.g., length, width, curvature), and detailed parameters, which define local morphological features (e.g., protrusion height, depression depth) (Abdul-Ghafour et al., 2014; Langerak, 2010).

The bionic fitting surface is generated by sweeping the bionic finger contour lines. Defining semantic feature parameters for this surface streamlines the instantiation of the attachment surface. This study focuses on the configuration of global semantic parameters, specifically those governing bionic fitting curvature and dimensional characteristics.

#### 2.3.1 Bionic fitting bending parameters

**FIGURE 4:**
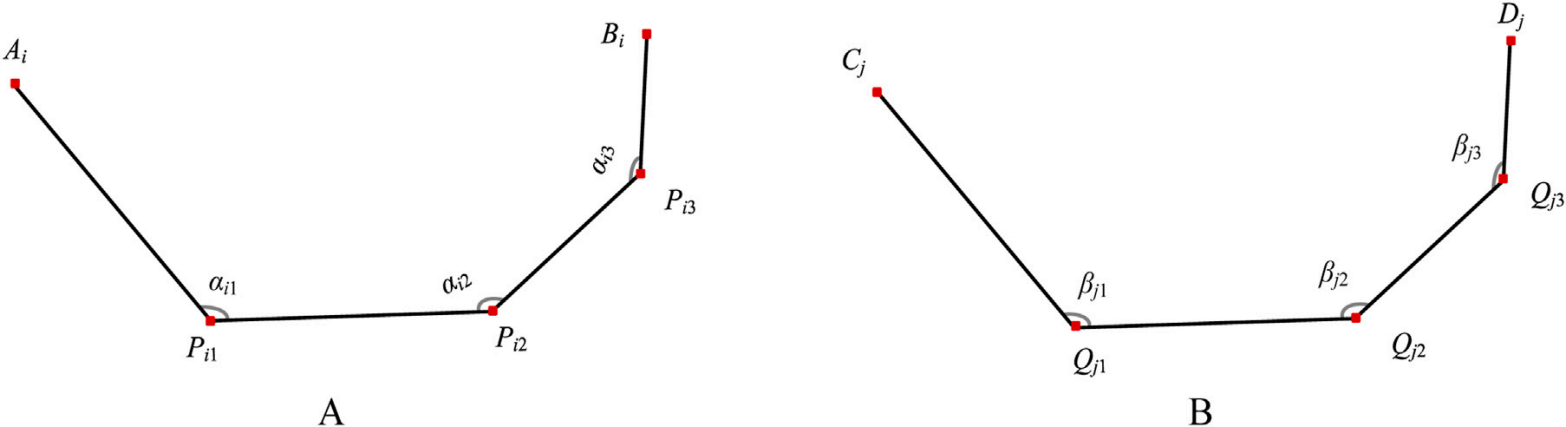
Bending angles of bionic finger contour feature lines. **(A)** Medial finger contour feature line (numbered *i*), comprising first-level key bending angle *α*_*i*1_, second-level key bending angle *α*_*i*2_, and third-level key bending angle *α*_*i*3_. **(B)** Lateral finger contour feature line (numbered *j*), comprising first-level key bending angle *β*_*j*1_, second-level key bending angle *β*_*j*2_, and third-level key bending angle *β*_*j*3_.

The bionic fitting bending parameters are determined by the finger key bending angles of the bionic hand-inspired structure. As shown in Figure 4, each finger contour line has three key bending angles: the first-level key bending angle (*α*_*i*1_), second-level key bending angle (*α*_*i*2_), and third-level key bending angle (*α*_*i*3_). The first-level key bending angle (*α*_*i*1_) is the angle between *A*_*i*_ *P*_i1_ and *P*_i1_ *P*_i2_. The second-level key bending angle (*α*_*i*2_) is the angle between *P*_*i*1_ *P*_*i*2_ and *P*_*i*2_ *P*_*i*3_. The third-level key bending angle (*α*_*i*3_) is the angle between *P*_*i*2_ *P*_*i*3_ and *P*_*i*3_ *B*_*i*_. The bending angles are calculated using Equation 3.

$$\left\{\begin{array}{c} \alpha_{i1}=\arccos\left(\frac{\vec{P_{i1}A_{i}}\cdot \vec{P_{i1}P_{i2}}}{\left|\vec{P_{i1}A_{i}}\right|\times \left|\vec{P_{i1}P_{i2}}\right|}\right)\\ \alpha_{i2}=\arccos\left(\frac{\vec{P_{i2}P_{i1}}\cdot \vec{P_{i2}P_{i3}}}{\left|\vec{P_{i2}P_{i1}}\right|\times \left|\vec{P_{i2}P_{i3}}\right|}\right)\\ \alpha_{i3}=\arccos\left(\frac{\vec{P_{i3}P_{i2}}\cdot \vec{P_{i3}B_{i}}}{\left|\vec{P_{i3}P_{i2}}\right|\times \left|\vec{P_{i3}B_{i}}\right|}\right) \end{array}\right.$$ (3)

The lateral bionic fitting bending parameters are similarly defined. The first-level key bending angle (*β*_*j*1_) is the angle between *C*_*j*_ *Q*_*j*1_ and *Q*_*j*1_ *Q*_*j*2_. The second-level key bending angle (*β*_*j*2_) is the angle between *Q*_*j*1_ *Q*_*j*2_ and *Q*_*j*2_ *Q*_*j*3_. The third-level key bending angle (*β*_*j*3_) is the angle between *Q*_*j*2_ *Q*_*j*3_ and *Q*_*j*3_ *D*_*j*_. The bending angles are calculated using Equation 4.

$$\left\{\begin{array}{c} \beta_{j1}=\arccos\left(\frac{\vec{Q_{j1}C_{j}}\cdot \vec{Q_{j1}Q_{j2}}}{\left|\vec{Q_{i1}C_{j}}\right|\times \left|\vec{Q_{j1}Q_{j2}}\right|}\right)\\ \beta_{j2}=\arccos\left(\frac{\vec{Q_{j2}Q_{j1}}\cdot \vec{Q_{j2}Q_{j3}}}{\left|\vec{Q_{j2}Q_{j1}}\right|\times \left|\vec{Q_{j2}Q_{j3}}\right|}\right)\\ \beta_{j3}=\arccos\left(\frac{\vec{Q_{j3}Q_{j2}}\cdot \vec{Q_{j3}D_{j}}}{\left|\vec{Q_{j3}Q_{j2}}\right|\times \left|\vec{Q_{j3}D_{j}}\right|}\right) \end{array}\right.$$ (4)

#### 2.3.2 Bionic fitting size parameters

##### 2.3.2.1 Length parameters

**FIGURE 5:**
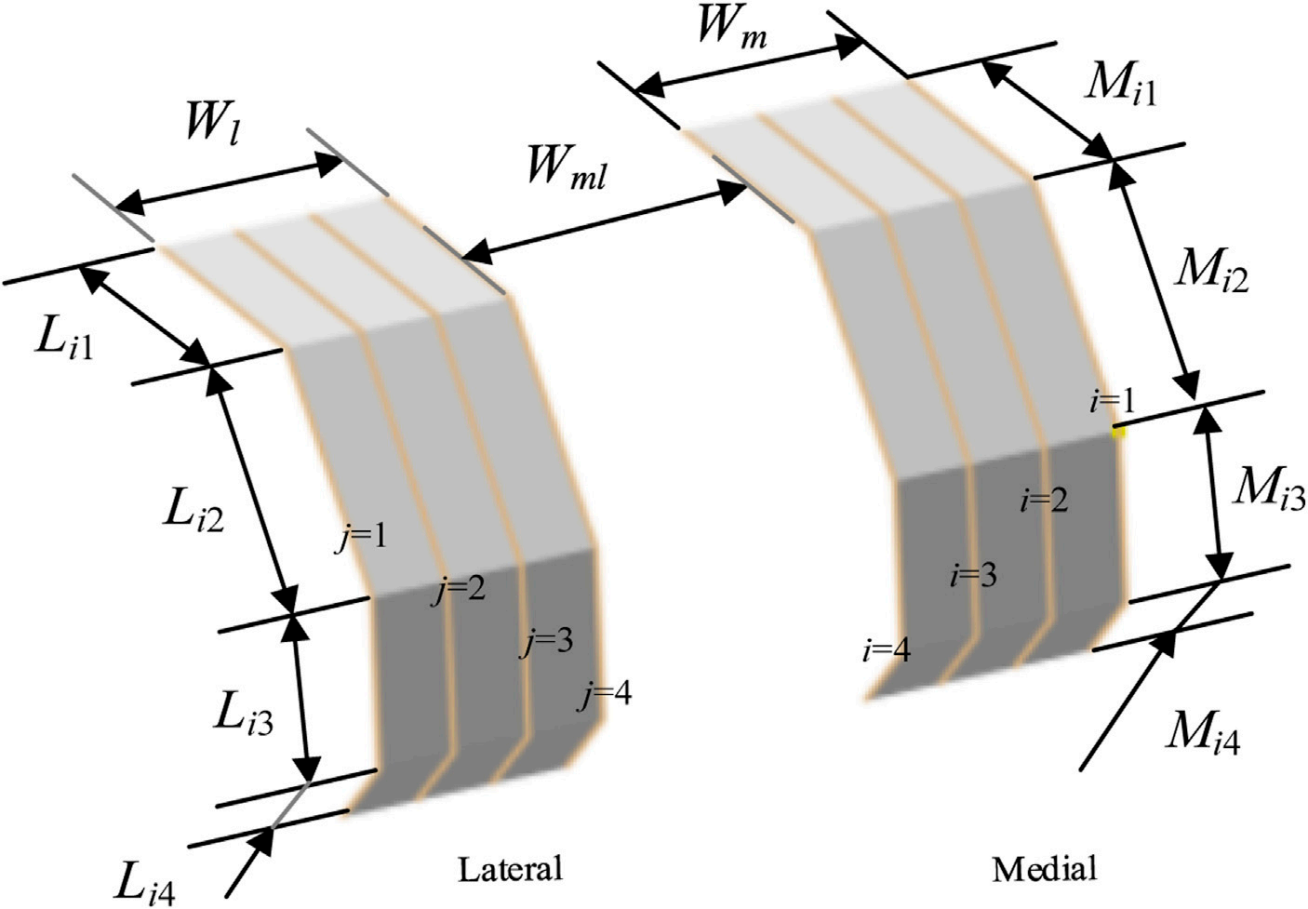
Configuration of bionic fitting size parameters, encompassing length parameters and width parameters.

As shown in Figure 5, for any finger contour feature line numbered *i*, the length parameters are set as follows: the length of *A*_*i*_ *P*_i1_ (*M*_i1_) is determined by *d*_0_ and *α*_*i*1_; the length of *P*_i1_ *P*_i2_ (*M*_i2_) is the distance between *P*_*i*1_ and *P*_*i*2_; the length of *P*_*i*2_ *P*_*i*3_ (*M*_*i*3_) is the distance between *P*_*i*2_ and *P*_*i*3_; the length of *P*_*i*3_ *B*_*i*_ (*M*_*i*4_) is determined by *d*_0_ and *α*_i3_. The equation is defined as follows Equation 5.

$$\left\{\begin{array}{c} \begin{array}{c} M_{i1}=\left|\vec{{A_{i}P}_{i1}}\right|==\frac{d}{\cos\left(\alpha_{i1}‐\frac{\pi }{2}\right)}\\ M_{i2}=\left|\vec{{P_{i1}P}_{i2}}\right| \end{array}\\ \begin{array}{c} M_{i3}=\left|\vec{{P_{i2}P}_{i3}}\right|\\ M_{i4}=\left|\vec{{P_{i3}B}_{i}}\right|=\frac{d}{\cos\left(\alpha_{i3}‐\frac{\pi }{2}\right)} \end{array} \end{array}\right.$$ (5)

Similarly, for any finger contour feature line numbered *j*, the length parameters are set as follows: the length of *C*_*j*_ *Q*_*j*1_ (*L*_*j*1_) is determined by *d*_0_ and *β*_*j*1_; the length of *Q*_*j*1_ *Q*_*j*2_ (*L*_j2_) is the distance between *Q*_*j*1_ and *Q*_*j*2_; the length of *Q*_*j*2_ *Q*_*j*3_ (*L*_*j*3_) is the distance between *Q*_*j*2_ and *Q*_*j*3_; the length of *Q*_*j*3_ *D*_*j*_ (*L*_j4_) is determined by *d*_0_ and *β*_*j*3_. The model is defined by Equation 6.

$$\left\{\begin{array}{c} \begin{array}{c} L_{j1}=\left|\vec{{C_{j}Q}_{j1}}\right|==\frac{d}{\cos\left(\beta_{j1}‐\frac{\pi }{2}\right)}\\ L_{j2}=\left|\vec{{Q_{j1}Q}_{j2}}\right| \end{array}\\ \begin{array}{c} L_{j3}=\left|\vec{{Q_{j2}Q}_{j3}}\right|\\ L_{j4}=\left|\vec{{Q_{j3}D}_{j}}\right|=\frac{d}{\cos\left(\beta_{j3}‐\frac{\pi }{2}\right)} \end{array} \end{array}\right.$$ (6)

##### 2.3.2.2 Width parameters

As shown in Figure 5, the width parameters of the bionic fitting surface include the following: the medial bionic fitting surface width (*W*_m_), equivalent to the projection distance of $\vec{M_{\text{medial}}M_{\text{lateral}}}$ onto the coronal plane; the lateral bionic fitting surface width (*W*_l_), equivalent to the projection distance of $\vec{L_{\text{medial}}L_{\text{lateral}}}$ onto the coronal plane; the distance between the medial and lateral bionic palms (*W*_ml_), equivalent to the width of the intercondylar fossa; the height of the medial bionic fitting surface (*H*_m_), equivalent to the projection distance of $\vec{M_{\text{high}}M_{\text{low}}}$ onto the sagittal plane; and the lateral bionic fitting surface height (*H*_l_), equivalent to the projection distance of $\vec{L_{\text{high}}L_{\text{low}}}$ onto the sagittal plane.

## 3 Results and discussion

The femoral CT data of a 50-year-old Han Chinese male patient, with a height of 175 cm, were selected for this study. A Light Speed VCT helical scanner (GE Healthcare) was employed for data acquisition. The experiments were conducted on a Windows-10 platform, equipped with an Intel^®^ Core™ i5-8th generation processor running at 2.30 GHz and 16 GB of memory.

### 3.1 Generation of bionic fitting surface

Feature lines serve as the fundamental units for surface modeling, with the surface shape being jointly determined by the configuration of these feature lines and the surface generation method employed. In this study, we utilized feature lines in conjunction with a specific surface generation technique to reconstruct bionic fitting surfaces. These surfaces encompass both medial and lateral aspects, with the medial bionic fitting surface conforming to the medial femoral condyle and the lateral bionic fitting surface adhering to the lateral femoral condyle.

**FIGURE 6:**
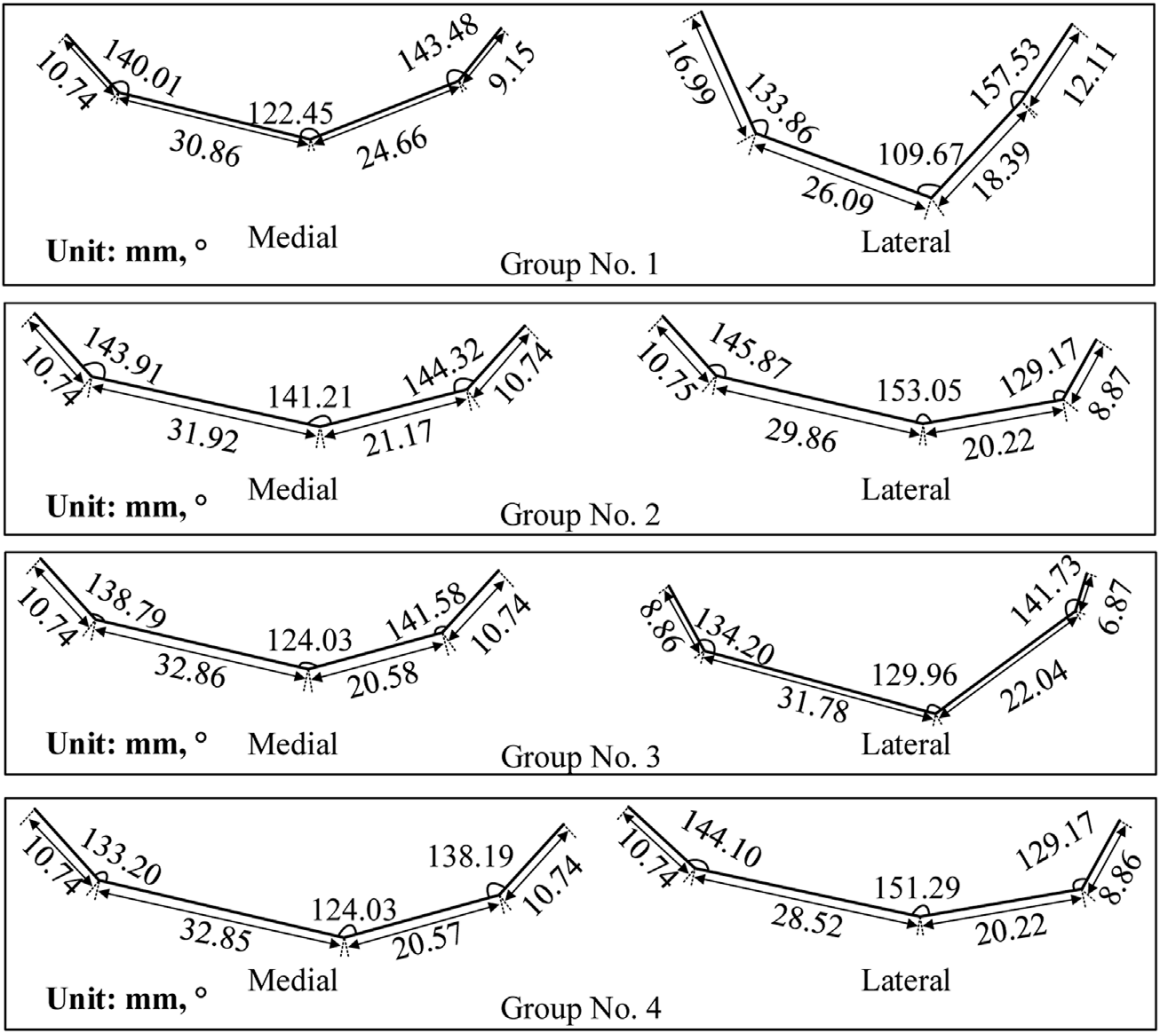
Finger contour feature lines of four experimental groups. Each group corresponds to a unique set of parameters presented in Table 1.

**FIGURE 7:**
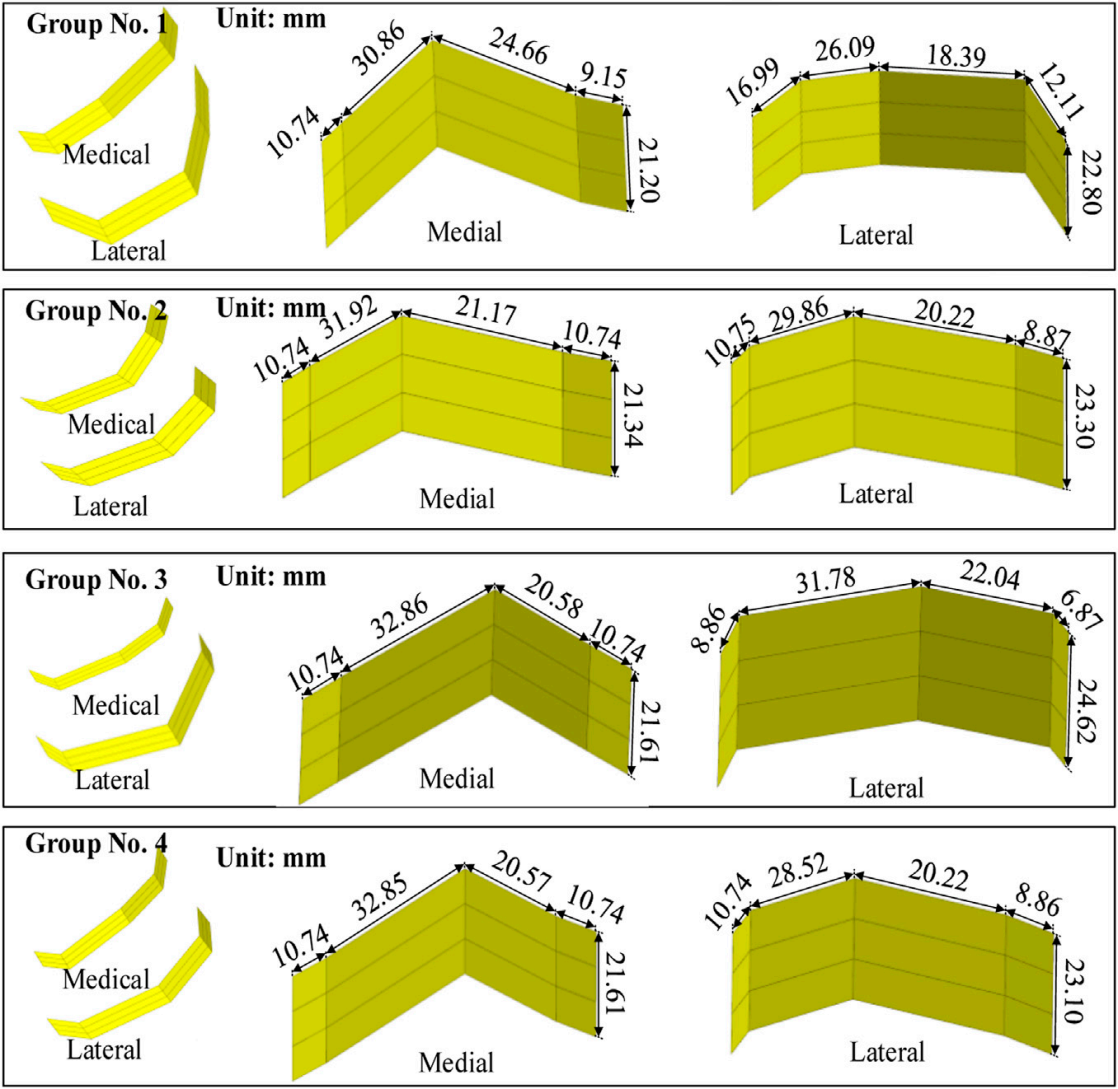
Femoral condylar bionic fitting surfaces. Comprising both medial and lateral surfaces, these fitting surfaces were constructed based on the finger contour feature lines illustrated in Figure 6. Each experimental group corresponds to a distinct parameter set outlined in Table 1 and visualized in Figure 6.

The adjustment coefficients ( δ _1_, δ _2_, δ _3_ in Formula (1) and ε _1_, ε _2_, ε _3_ in Formula (2)) were empirically calibrated through an iterative optimization process using a set of 20 femur CT scans, as detailed in Section 2.2.5, yielding a value of approximately 0.76. The bionic fitting surfaces and attachment surfaces presented in the following sections were generated using this initial value, successfully demonstrating the feasibility and precision of the parametric design method. The specific bending and size parameters of the bionic fitting surface are detailed in Table 1. The contour of the finger is visually represented in Figure 6, while the generated bionic fitting surface is depicted in Figure 7.

**FIGURE 8:**
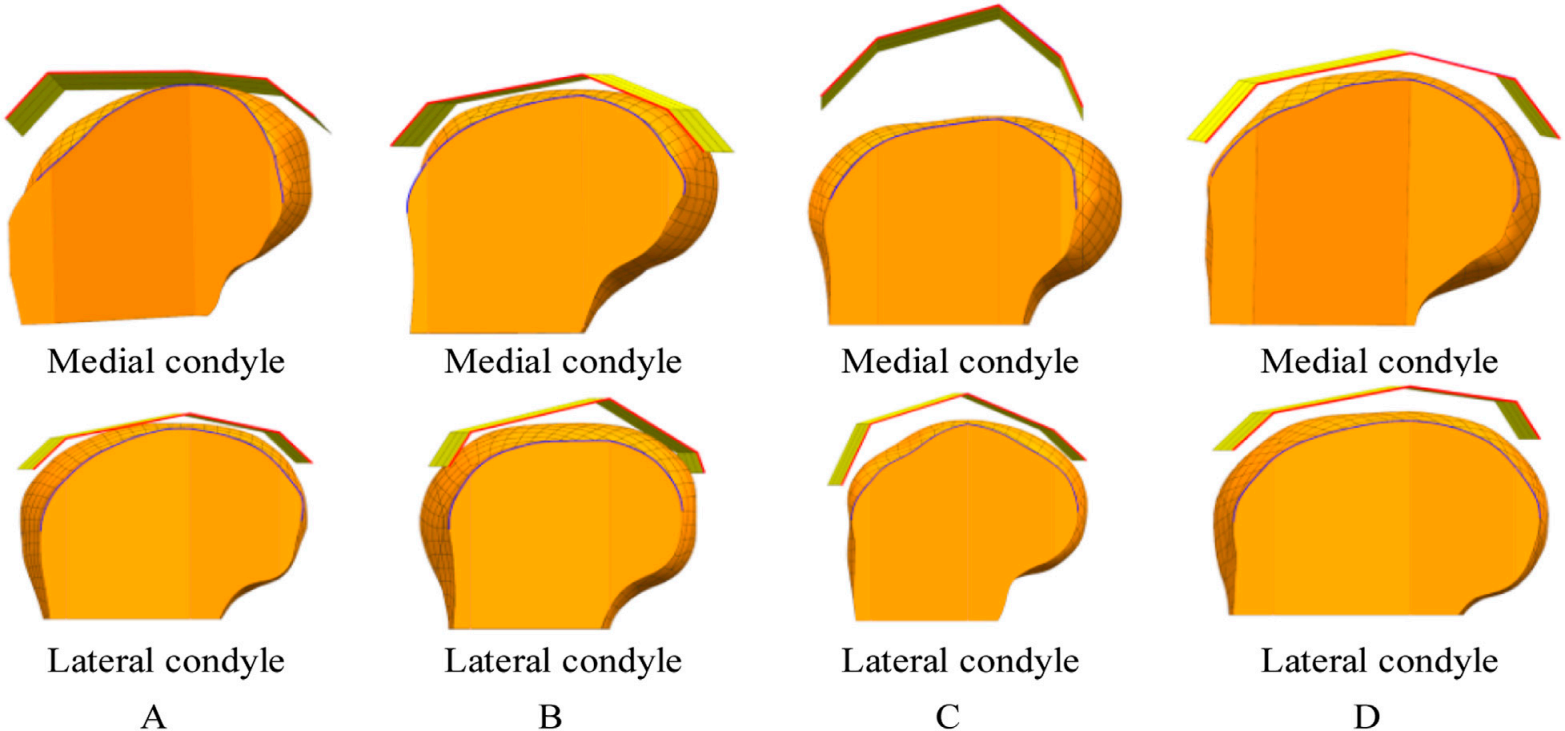
Projection lines generated by projecting the contour feature line (from Figure 7) onto the femoral condyle. The bionic palm attachment surface is projected onto the medial condyle of the femur. The red line represents the index finger contour feature line of the bionic palm attachment surface, while the blue line denotes the corresponding projection line on the femoral condyle. **(A)** Group No. 1. **(B)** Group No. 2. **(C)** Group No. 3. **(D)** Group No. 4.

**FIGURE 9:**
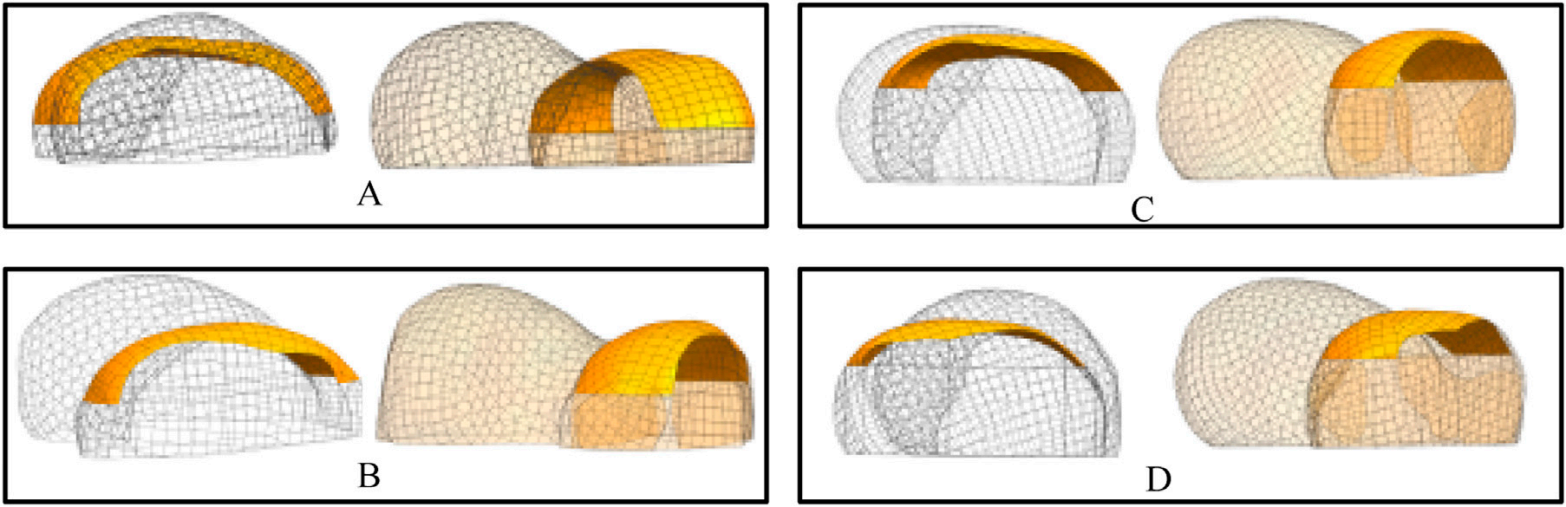
Lateral attachment surfaces. Each set of results provides a rendering of two viewpoints. **(A)** Attachment surfaces corresponding to the parameter sets in Table 1 (Group 1). **(B)** Attachment surfaces corresponding to the parameter sets in Table 1 (Group 2). **(C)** Attachment surfaces corresponding to the parameter sets in Table 1 (Group 3). **(D)** Attachment surfaces corresponding to the parameter sets in Table 1 (Group 4).

The procedure for constructing the attachment surface of the femoral condylar prosthesis involves several key steps. Initially, the contour line is obtained by projecting the bionic hand-inspired structure line onto the femoral condyle surface, as illustrated in Figure 8. Subsequently, the source surface is derived by segmenting the femoral condyle surface using these contour lines. Finally, a novel surface is reconstructed employing a surface generation method (filling), which serves as the femoral attachment surface. The lateral attachment surface is visually represented in Figure 9.

### 3.2 Fit analysis

#### 3.2.1 Hausdorff distance-based fit assessment

To rigorously verify the fit, we employed the Hausdorff distance (van Kreveld et al., 2022; Ellen et al., 2011), a metric renowned for its efficacy in evaluating surface reconstructions. Fit was quantitatively assessed by calculating the distance between the feature point set on the attachment surface of the femoral condylar prosthesis and the corresponding feature point set on the surface of the femoral condyle. Specifically, the set of feature points collected on the attachment surface (plate) was denoted as *A*_plate_, while the set of feature points collected on the femoral condyle surface was denoted as *B*_femur_. In the acquisition process, we primarily focused on collecting points that reflect the surface characteristics, including those on the finger contour feature line and the internal auxiliary feature line. The Hausdorff distance between the sets *A*_plate_ and *B*_femur_, denoted as *H* (*A*_plate_, *B*_femur_), is mathematically expressed as follows:

The Hausdorff distance between the sets *A*_plate_ and *B*_femur_, denoted as *H* (*A*_plate_, *B*_femur_), is defined by Equation 7.

$$H\left(A_{\text{plate}},B_{\text{femur}}\right)=\max\left\{\max_{a\in A_{\text{plate}}}\left\{\min_{b\in B_{\text{femur}}}\left\|a-b\right\|\right\},\max_{b\in B_{\text{femur}}}\left\{\min_{a\in A_{\text{plate}}}\left\|b-a\right\|\right\}\right\}$$ (7)

The point clouds were sampled at a density of 10 points/mm^3^ to ensure accurate representation of the surface curvature. It is worth noting that the clinical acceptability threshold of 0.5 mm for interface micromotion, derived from foundational studies on bone ingrowth (Pilliar et al., 1986), is a recognized benchmark for cementless femoral components (Springer et al., 2009). The mean Hausdorff distance achieved in this study (0.29 ± 0.03 mm) is significantly below this threshold, indicating high geometric fidelity. Potential sources of measurement error include CT image resolution and surface reconstruction algorithms, but the low coefficient of variation (10.3%) across parametric groups suggests the method is robust against these variations.

The experimental validation, resulting in a Hausdorff distance of 0.29 mm, confirms that the proposed design achieves high anatomical alignment precision while reducing design complexity compared to conventional CAD-based approaches. The key innovation of this work is its functional biomechanical adaptation, enabled by a hierarchical curvature control framework. This framework facilitates dynamic conformity to the femoral condyle anatomy by integrating three technical aspects: (1) proximal condyle apex mapping for longitudinal curvature modulation, (2) intercondylar fossa alignment for transverse bending adaptation, and (3) distal phalangeal parameterization for condylar base curvature optimization. The achieved precision of 0.29 mm validates this tri-level mapping mechanism, demonstrating the ability of the prosthetic surface to conform dynamically to the underlying bone. By translating principles of biological adaptation into a clinical design methodology, this work establishes a practical approach for enhancing implant-bone integration and shows potential for improving functional outcomes through patient-specific optimization.

The achieved sub-millimeter precision is a critical prerequisite for reducing micromotion and stress shielding, which are key biomechanical factors in long-term implant stability (Pilliar et al., 1986; Springer et al., 2009). However, it is important to note that superior geometric fit, while necessary, must be complemented by biomechanical validation to fully ascertain its clinical impact.

#### 3.2.2 Impact of model accuracy on fit

The geometric fidelity of the femoral bone model directly influences the resulting fit of the attachment surface. To investigate this relationship, we evaluated two mesh resolutions with explicit consideration of computational efficiency:

1. Mesh Configuration & Computational Load

Coarse mesh (8 mm element size, 791 elements total/183 condyle) was generated in 2.1 min on a standard workstation, while the fine mesh (3 mm element size, 5,990 elements total/1,337 condyle) required 18.7 min.

**FIGURE 10:**
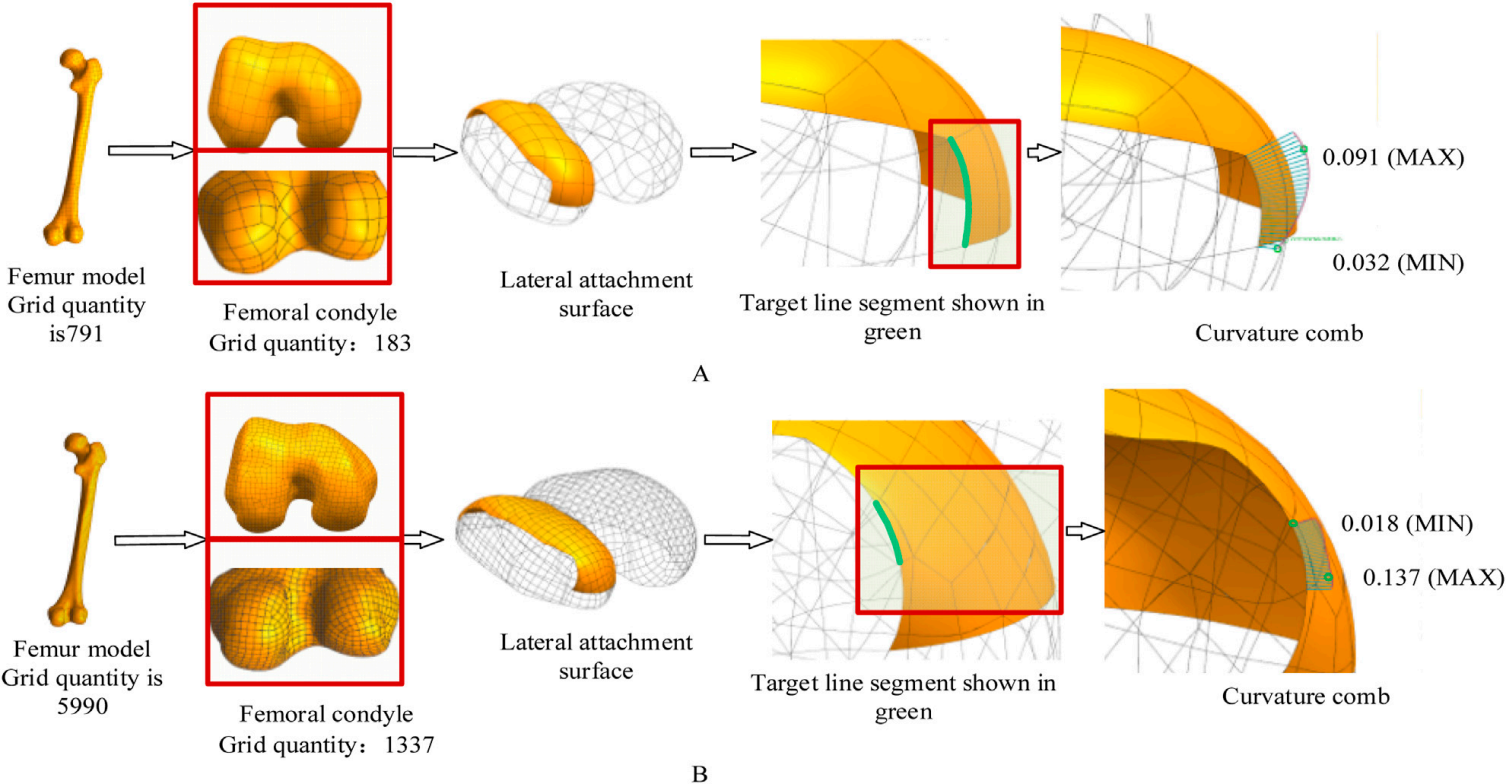
The effect of different mesh accuracies on the experimental results. In this experiment, the femoral mesh model is represented by a quadrangle mesh. The curvature comb is used to display the curvature direction change and magnitude of the contour feature line. The change of the curvature comb line in Figure 10B is more uniform, indicating better curve fairness. Maximum curvature decreased from 0.091 mm^-1^ **(A)** to 0.018 mm^-1^ **(B)**. **(A)** Coarse mesh (8 mm element size). **(B)** Fine mesh (3 mm element size).

Curvature analysis (Figure 10) revealed that the fine-mesh model achieved a 62% improvement in curvature transition smoothness (quantified by a reduction in the standard deviation of curvature along the profile from 0.012 mm^-1^ to 0.0045 mm^-1^) with reduced curvature comb fluctuations over the coarse-mesh model, but demanded 9× longer computation time, establishing a clear precision-efficiency tradeoff where coarse mesh suffices for preliminary *δ*/*ε* coefficient calibration whereas fine mesh is essential for final anatomical adaptation.

2. Practical Balancing Strategy

For clinical deployment, we recommend a two-tier approach: initial coefficient calibration using coarse mesh (≤8 mm) for rapid iteration in pilot cases, followed by patient-specific optimization with fine mesh (≤3 mm) and validated *δ*/*ε* coefficients. This hierarchical workflow reduces total computation time by 73% while maintaining Hausdorff distance <0.35 mm across 20 retrospective cases, with coefficient variations remaining below 5%.

### 3.3 Comparative analysis with state-of-the-art biomimetic designs

The core innovation of this work lies in its functional biomimicry. Unlike approaches that directly copy anatomical shapes, our method abstracts the hierarchical curvature control observed in the palmar-phalangeal system (Bai et al., 2024) into a parametric design system. This allows for dynamic adaptation to the femoral condyle’s surface, addressing the static geometric mismatch common in traditional CAD. Our bionic curvature-adaptation approach provides a new paradigm in orthopedic implant design by synergizing anatomical fidelity with clinical efficiency, as evidenced by these critical differentiators from leading methodologies shown in Table 2.

**TABLE 2:** quantifies these advances across four clinical dimensions.

Design dimension	Liu et al.	Herr et al.	Our method
Biological Basis	Data-driven biomechanics	Neuromuscular emulation	Palmar-phalangeal functional hierarchy
Clinical workflow	Pre-op scan → ML/FEA	Robotic calibration	Intraoperative parametric editing
Accuracy validation	0.25 mm surface RMSE	N/A	0.29 mm Hausdorff distance
Technical Complexity	Python/FEA expertise required	Robotic integration needed	Minimal training required (surgeon-accessible parameters)

1. Versus data-driven biomechanical optimization (Liu et al., 2022):

While Liu’s framework achieves high accuracy (0.25 mm surface error) through machine learning and FEA, it mandates preoperative CT/MRI scans and extensive computations. Our parametric system enables intraoperative real-time fitting using intuitive bending/size parameters, eliminating imaging dependencies while maintaining clinically equivalent precision (0.29 mm Hausdorff distance).

2. Versus robotic functional augmentation (Herr et al., 2023):

Herr’s ankle-foot emulator excels in dynamic terrain adaptation via torque-controlled actuation but requires complex mechatronic integration. Our focus on static morphological conformity avoids hardware dependencies and surgical risks, directly addressing TKR’s core need for anatomical precision.

### 3.4 Translational pathways for future validation and clinical integration

The proposed parametric framework establishes a clear pathway for clinical translation, although empirical fabrication and biomechanical testing are beyond the scope of this methodological study. The generated attachment surfaces (Figure 9) are compatible with orthopedic additive manufacturing techniques, such as electron beam melting of Ti-6Al-4V (Liu and Shin, 2019), as their continuous curvature profiles avoid complex undercuts. Crucially, the parametric outputs of this method provide direct input for future finite element analysis (FEA). The semantically defined parameters (e.g., bending angles *α*, *β*) allow for the systematic generation of models with controlled geometrical variations. This enables computational benchmarking to quantitatively evaluate biomechanical performance, such as quantifying the reduction in stress-shielding at the bone-implant interface compared to traditional CAD-based designs—a key step in predicting long-term stability.

The parametric nature of this approach promises to enhance the clinical workflow. By integrating with surgical planning software, it could significantly reduce preoperative design time compared to labor-intensive CAD methods. The parameters (e.g., bending angles, sizes) are designed to be intuitive, potentially facilitating intraoperative customization by surgeons, though future work must evaluate its intra- and inter-operator reproducibility.

Looking forward, this methodology can be positioned within the broader trend of data-driven orthopedic innovation. The integration of emerging technologies, such as kinematic sensors for postoperative mobility and load assessment (Di Puccio et al., 2025), could provide invaluable *in-vivo* data to refine design parameters based on actual patient activity. Similarly, insights from optimized rehabilitation protocols could inform the design goals to better support postoperative recovery. This creates a closed-loop system connecting design, implantation, functional outcome, and rehabilitation, ultimately paving the way for adaptive, patient-specific implants that are biomechanically efficient and rehabilitation-ready.

### 3.5 Limitations and future work

This study presents a novel methodological framework, and its validation was primarily conducted on a single representative case to demonstrate feasibility and precision. While the parametric variations (n = 4 groups) showcased the adaptability of the design, this approach limits the generalizability of the results across diverse patient populations with varying femoral morphologies (e.g., different genders, ages, and ethnicities). Furthermore, the validation at this stage is geometric; the biomechanical performance of the designed surfaces remains to be thoroughly investigated.

Future work will focus on three critical directions to address these limitations and advance the technology:

First, biomechanical validation will be prioritized. The parametric models generated in this study will be directly used in finite element analysis to simulate interface stresses and micromotion under physiological loading conditions, providing a computational assessment of their performance advantage. Subsequent stages will include prototype fabrication and mechanical testing under simulated physiological loads to assess fatigue life and interfacial stability.

Second, a comprehensive validation on a larger, demographically diverse cohort will be conducted to statistically calibrate the design parameters and establish population-wide applicability.

Finally, the potential of this bionic parametric approach will be explored for other orthopedic implants, such as tibial components and acetabular cups, to validate its broader utility.

## 4 Conclusion

This study introduced a novel parametric design methodology for femoral condylar prosthesis attachment surfaces, inspired by the multi-level curvature adaptation mechanism of the human hand. The core contribution lies in translating the functional hierarchy of the palmar-phalangeal system into a parametric design framework, moving beyond simple shape replication to enable dynamic anatomical fitting.

The proposed method offers two significant advantages: (1) It constructs a bionic fitting surface through semantically meaningful parameters (bending angles and sizes), which significantly enhances design flexibility and efficiency compared to traditional CAD-based approaches. (2) It generates an attachment surface that achieves exceptional anatomical conformity, as evidenced by a Hausdorff distance of 0.29 mm, promising improved prosthetic support and fit.

It is important to acknowledge the limitations of this study, primarily its validation on a single representative case, which affects the generalizability of the results. Furthermore, the current validation is geometric, and biomechanical performance remains to be assessed.

Future work will focus on three critical directions: First, expanding the validation to larger and more demographically diverse cohorts to ensure broad applicability. Second, conducting comprehensive biomechanical evaluations, including finite element analysis to quantify stress shielding and experimental testing under simulated physiological loads to assess micromotion and long-term stability. Finally, the potential of this bionic parametric approach will be explored for other orthopedic implants, such as tibial components and acetabular cups, and its integration with postoperative monitoring technologies and rehabilitation strategies will be investigated to close the loop between design, implantation, and functional recovery.

## Data Availability

The original contributions presented in the study are included in the article/supplementary material, further inquiries can be directed to the corresponding author.
